# Effects of sumac supplementation on metabolic markers in adults with metabolic syndrome: a triple-blinded randomized placebo-controlled cross-over clinical trial

**DOI:** 10.1186/s12937-023-00854-9

**Published:** 2023-05-15

**Authors:** Fateme Sadat Mirenayat, Zahra Hajhashemy, Mansour Siavash, Parvane Saneei

**Affiliations:** 1 Student Research Committee, Isfahan University of Medical Sciences, Isfahan, Iran; 2 Department of Community Nutrition, School of Nutrition and Food Science, Nutrition and Food Security Research Center, Isfahan University of Medical Sciences, PO Box 81745-151, Isfahan, Iran; 3 Endocrine and Metabolism Research Center, Isfahan University of Medical Sciences, Isfahan, Iran

**Keywords:** Sumac, Supplementation, Metabolic syndrome, Randomized clinical trial

## Abstract

**Background:**

Despite the prior evidence of the impacts of sumac on glycemic indices, lipid profile and visceral fat, there is a lack of evidence regarding the efficacy of sumac in cases with metabolic syndrome (MetS). Therefore, we aimed to assess the effect of sumac supplementation on MetS markers among adults with this syndrome.

**Methods:**

In this triple-blinded randomized placebo-controlled cross-over clinical trial 47 adults with MetS were randomly assigned to receive 500 mg sumac or placebo (lactose) capsule, twice a day. Each phase took 6 weeks and there was a 2-week washout between phases. All clinical evaluations and laboratory tests were conducted before and after each phase.

**Results:**

At the baseline of the study, mean (± SD) age, weight, and waist circumference of participants were respectively 58.7 (± 5.8) yr, 79.9 (± 14.3) kg, and 107.6 (± 10.8) cm. Intention to treat analysis (ITT) analyses revealed that sumac supplementation decreased systolic blood pressure by 5 mmHg (128.8 ± 21.4 at the baseline vs. 123.2 ± 17.6 after 6 weeks intervention, *P* = 0.001). The comparison of changes in two trial arms showed that sumac supplementation significantly reduced systolic blood pressure (sumac group -5.59 ± 10.6 vs. control group 0.76 ± 10.5, *P* = 0.004), but did not change anthropometric indices or diastolic blood pressure. Similar results were also found in the per-protocol analyses.

**Conclusions:**

This cross-over trial revealed that sumac supplementation could reduce systolic blood pressure in men and women with MetS. Daily intake of 1000 mg sumac, as an adjuvant therapy, may be beneficial in management of MetS in adults.

## Introduction

*Rhus Coriaria* (RC), commonly known as sumac, is a seasoning, spice, flavoring agent, or condiment, especially used in Middle Eastern and Mediterranean countries [1, 2]. Sumac, which has been used as folk medicine since ancient times, is rich in biologically active substances (such as flavonoids, flavones, phenolic acids, hydrolysable tannins, quercetin and anthocyanin) for improving cardiovascular health [1]. Prior investigations have proposed several beneficial activities such as antimicrobial, antiviral, anti-inflammatory, antioxidant, and blood glucose lowering for sumac. Additionally, sumac could have a protective effect against liver damage via free oxygen radical-scavenging mechanism. Anti-hemolytic and anti-fibrogenic properties have also been suggested for this herb [2].

Both experimental investigations on animals and randomized clinical trials (RCTs) on human subjects have demonstrated favorable effects of sumac supplementation on metabolic markers [2–11]. Sumac could improve lipid profile in hyperlipidemic mice [11] and patients with mild to moderate hyperlipidemia [1]. Among overweight/obese women with depression, sumac supplementation along with a restricted calorie diet could significantly reduce weight, body mass index (BMI), body and visceral fat, and malondialdehyde (MDA) levels [3]. Daily intake of sumac in diabetic patients could also result in significant declines in insulin levels, homeostatic model assessment of insulin resistance (HOMA-IR), MDA, and high sensitive C-reactive protein (hs-CRP) [4]. Despite the prior evidence of the impacts of sumac on glycemic indices, lipid profile and visceral fat, there is a lack of evidence regarding the efficacy of sumac in cases with metabolic syndrome (MetS). This syndrome is a combination of several disorders including high blood pressure, dyslipidemia, abdominal obesity and high blood glucose [12–14]. MetS has recently become a public health threat [15, 16] and about 25 to 35 percent of adults around the world suffer from this disorder [17]. Since no single appropriate treatment has been identified for treatment of MetS and taking chemical drugs might have several adverse side effects [18], there has been a great interest in finding natural substances to treat and manage MetS [19]. Therefore, the present study aimed to assess the effect of sumac supplementation on MetS components among adults with this syndrome.

## Methods

### Participants

A single-center triple-blind randomized placebo-controlled cross-over clinical trial was performed in Isfahan Endocrine and Metabolic Research Center (IEMRC) between November 2020 and June 2021. Individuals with MetS with the age range of 20 to 70 years who were willing to participate in the trial were included in the current analysis. According to the National Cholesterol Education Program Adult Treatment Panel III (NCEP/ATP III) definition [20], MetS was defined as having three or more of the following criteria: 1) large waist circumference (women > 88 and men > 102 cm); 2) high triglyceride level (TG ≥ 150 mg/dl); 3) low high density lipoprotein-cholesterol (HDL-c) concentrations (women < 50 and men < 40 mg/dL); 4) high systolic and/or diastolic blood pressure (SBP ≥ 130 and/or DBP ≥ 85 mmHg) and 5) high fasting blood sugar (FBS ≥ 100 mg/dL). Those with the following criteria were not include in this trial: 1) having clinical history of the following disease: cardiovascular, liver, kidney, thyroid, stroke and diabetes mellitus; 2) being pregnant or lactating; 3) following special dietary patterns; 4) taking medications that affect appetite, blood pressure, inflammation, lipid or glycemic profile, fat or carbohydrate metabolism; 5) using multivitamin-mineral supplements, omega-3 fatty acids or herbal remedies; and 6) having covid-19 infection. By the use of the standard formula suggested for two-period, two-treatment cross-over studies and considering a power of 80% to detect the difference of at least 5 mg/dL in mean FBS (as a key dependent variable), type I error of 5%, and the standard deviation of 13.75 mg/dL for FBS [1], the sample size was determined to be a total of 30 participants. Considering the high rate of drop out, due to cross-over design and high prevalence of covid-19 pandemic during the study implementation in Iran, 47 subjects were finally recruited in the current intervention. All methods were performed in accordance with the Declaration of Helsinki guidelines and regulations. A written informed consent was signed by each participant, before entering the study. This study was approved by the Medical Ethics Committee of Isfahan University (no.398797). The study protocol was registered at the Iranian Registry of Clinical Trials (www.IRCT.IR) under the registration number of IRCT20200106046022N1; 08/04/2020.

### Study design

Since the components of available sumac powder in the market might be non-standard, *Rhus coriaria* fruits were prepared from the medicinal herbs market, and were confirmed by two herbal botanists regarding taxonomical aspects. In order to have the most amounts of phenol acids and flavonoids of sumac (as the most effective gradients of this herb), the Folin–Ciocalteu method was used to standardize the powder of this herb based on its phenolic content [21]. The fruits were washed, dried and grinded. Then, the seeds were extracted from the powder by a sieve. After preparing sumac powder, each empty capsule was filled with 500 mg this powder for the intervention group. The same amount of lactose powder was used to fill the placebo capsules. The shape, color and size of sumac and placebo capsules were the same. Finally, the packages of sumac or placebo (containing 84 capsules) were provided for each phase. These packages were labeled as A and B. All patients and investigators were blinded to the supplementation type. Participants were randomly assigned to the intervention group (assigned to 500 mg sumac capsule, twice a day) or control group (assigned to 500 mg lactose capsule, twice a day). This study consisted of 2 phases (sumac and placebo arms) with a 2-week washout period between them. In order to have significant changes in blood glucose and lipid profiles, dose of sumac and duration of the study were determined based on previous studies [1, 22–24].

**Fig. 1:**
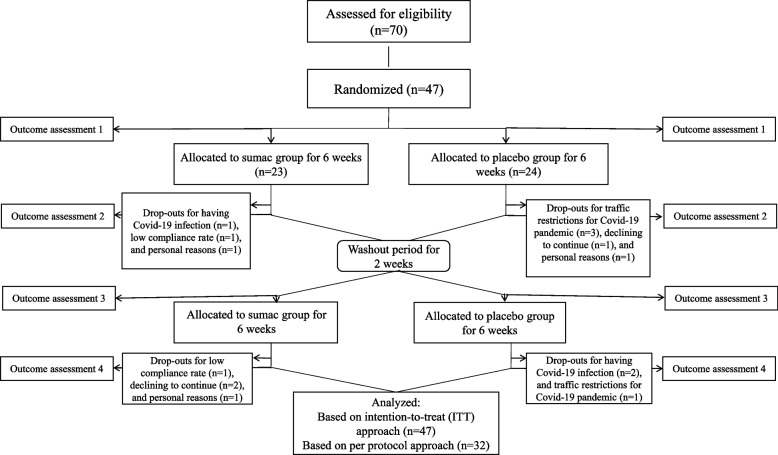
Participant flow diagram

At the initial of study, participants were randomly assigned to one of 2 groups. In order to have appropriate randomization and blinding, an unaware person, who was not involved in the study, performed randomization through the website of “www.randomization.com” and assigned a code to each participant. Thus, subjects were randomly divided into two groups of intervention and control with a 1:1 allocation ratio. Coding of sumac and placebo capsules with A or B was also performed by a person who was not involved in sampling, data collection and analysis. Therefore, all subjects and researchers were blinded to the randomization status and treatment assignment. An independent third party has supervised the blind process. In the first phase, patients of group 1 (*n* = 23) received sumac capsules and the patients of group 2 (*n* = 24) consumed placebo capsules, twice a day after lunch and dinner meal, for 6 weeks. In the washout period, treatment was stopped for 2 weeks. In the second phase, the groups were crossed over; such that, group 1 was supplemented with placebo and group 2 was supplemented with sumac, twice a day after lunch and dinner meal, for 6 weeks. In order to improve the adherence to the intervention, we reminded the participants -via weekly phone call- to use the capsules and counted the remaining capsules at each visit. At the end of the study, a total of 7 individuals were excluded from the sumac supplementation group due to covid-19 infection (*n* = 1), low compliance rate (*n* = 2), decline to continue (*n* = 2), and personal reasons (*n* = 2). Eight participants of placebo groups were also lost to follow up because of covid-19 infection (*n* = 2), traffic restrictions for covid-19 pandemic (*n* = 4), decline to continue (*n* = 1), and personal reasons (*n* = 1). There were no significant differences between lost to follow up from sumac and placebo arm. Participants reported no clinical adverse effect and no individuals were excluded because of an adverse event of supplementation. The details of follow-up process are presented in Fig. 1. Although some participants were excluded, all 47 participants were included in analysis of intention-to-treat (ITT) approach.

### Measurements

All clinical evaluations and laboratory tests were conducted before and after each phase. A trained nutritionist gathered the information about socio-demographic and medical history of participants through the face-to-face interview. Half way between the lower rib margin and the iliac crest was measured as waist circumference (WC), in centimeters. For weight measurement, individuals were weighed by a digital scale (OMRON, HN-286-E, Japan; with the accuracy of ± 100 g) without shoes, while wearing light clothes. Additionally, participants stood without shoes for height measurement, and a portable stadiometer (Seca, Hamburg, Germany, with accuracy of 0.5 cm) was used for this measurement. BMI was computed through weight (kg) divided by the square of the height (m^2^). Moreover, using a digital sphygmomanometer (OMRON, M3, HEM-7154-E, Japan; with accuracy of 0.5 mmHg), blood pressure of participants was measured twice in a sitting position. Before each BP measurement, participants were rested for 5 min. Finally, the average of two measurements was considered for each individual.

Subjects were recommended to maintain their normal diet and lifestyle throughout all study phases. An expert nutritionist instructed individuals to complete a 3-day food record in each phase for assessment of energy and dietary intakes. Participants completed these records during a week in both phases (sumac and placebo arms) for two weekdays and 1 weekend day. Then, using the Iranian household measures, dietary intakes were converted to gram/day [25]. For calculation of daily energy and nutrient intake, Nutritionist IV software (adapted from the US National Nutrient Databank) which was modified for Iranian foods was used. We also asked the participants to record their physical activities for two nonconsecutive days in each phase of the intervention. Considering the type, intensity and duration of activities, data of these physical activity records were then expressed as metabolic equivalent intensities (MET).

A 12-h fasting blood sample was gathered from each participant, before and after of each study phase. After centrifuging blood samples for 10 min at room temperature, serum fasting blood glucose (FBG) concentration was assessed on the day of blood collection by enzymatic colorimetric method and through the use of the glucose oxidase (Pars Azmoon commercial kits, Tehran, Iran). Commercial kits were used for measurement of serum triglyceride, low density lipoprotein-cholesterol (LDL-c), HDL-c and total cholesterol concentrations by direct enzymatic colorimetric method (Pars Azmoon commercial kits, Tehran, Iran) and using a biochemical auto analyzer (Alpha Classic, Sanjesh Company, Iran).

### Statistical analysis

The normality of each variable distribution was examined by the use of Kolmogorov–Smirnov test. Descriptive statistics (means, SDs or SEs and range) were used to describe general characteristics of the study participants. For comparison of baseline characteristics between study groups, a paired t-test was applied. Changes in each variable in sumac and placebo arms were calculated by subsidizing baseline values from values of 6^th^ week. A paired t-test was also used for within-group and between-group comparisons to examine the effect of sumac intervention on MetS markers. Since the current RCT had a cross-over design, each participant served as his or her own control. This issue could control the effect of covariates and the inter-subject variability from the comparison between groups. Therefore, no adjustment was done in the analyses. Due to dropping out some participants, both an ITT and a per-protocol analysis were conducted. For the ITT analysis, the last-observation-carried-forward (LOCF) method was applied and the last observations carried forward for those visits with unavailable data. For the per-protocol analysis, those participants who completed the interventions and all clinic visits, were only included in the analysis. SPSS 18 was utilized to conduct all statistical analyses (SPSS Inc., Chicago, IL, USA). P values less than 0.05 were considered as statistically significant.

## Results

**Fig. 2:**
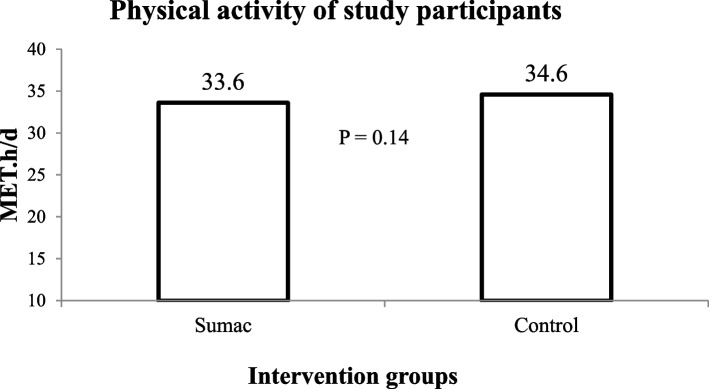
Physical activity levels of participants throughout the intervention arms

This cross-over RCT was conducted on 47 adults with MetS (81% women and 19% men). At the baseline of the study, mean (± SD) age, weight, waist circumference and BMI of participants were respectively 58.7 yr (± 5.8), 79.9 kg (± 14.3), 107.6 cm (± 10.8), and 31.6 kg/m^2^ (± 4.6), as shown in Table 1. Nutrient intakes of participants based on their 3-day dietary records during the intervention periods are provided in Table 2. No significant differences were observed between two groups of sumac and control in case of energy or macronutrients intakes. Other dietary intakes of participants, including saturated fatty acids, n-3 fatty acids, cholesterol, sodium, potassium, magnesium, calcium, folate, vitamin C and total dietary fiber were also not significantly different in the sumac and placebo arm. Physical activity levels of participants throughout the intervention periods are provided In Fig. 2. There was no significant difference in physical activity of participants between two intervention arms.

**Table 1:** Baseline characteristics of the study participants

	Mean	SD	Minimum	Maximum
Age (y)	58.7	5.83	42	69
Weight (kg)	79.9	14.35	58.7	117.4
Height (cm)	160	7.67	145	175
Body Mass Index (kg/m^2^)	31.6	4.6	23.3	42.7
Weight Circumference (cm)	107.6	10.86	88	131
Systolic blood pressure (mmHg)	129.7	21.5	93	183
Diastolic blood pressure (mmHg)	83.6	10.42	67	114

**Table 2:** Dietary intake of the study participants during intervention periods^a^

	Sumac group	Control group	P^b^
Energy (kcal)	1751.3 ± 141.5	1639 ± 88.1	0.51
Carbohydrates (% of E)	55.5 ± 1.6	55.0 ± 1.2	0.79
Proteins (% of E)	17.17 ± 0.75	18.27 ± 0.78	0.24
Fats (% of E)	30.3 ± 1.3	30.1 ± 1.1	0.91
Saturated fatty acids (g/d)	12.9 ± 1.8	11.9 ± 0.8	0.63
Cholesterol (mg/d)	173.1 ± 23.2	193.4 ± 21.6	0.47
Sodium (mg/d)	2017.7 ± 246.4	1848.9 ± 97.5	0.50
Potassium (mg/d)	2395.5 ± 187.8	2159.9 ± 144.6	0.33
Magnesium (mg/d)	311.9 ± 27.3	297.2 ± 17.4	0.63
Calcium (mg/d)	544.6 ± 59.4	498.1 ± 40.1	0.52
Folate (mg/d)	268.5 ± 22.9	276.0 ± 21.6	0.83
Vitamin C (mg/d)	129.3 ± 16.8	118.9 ± 11.7	0.61
Dietary fiber (g/d)	26.4 ± 2.3	27.4 ± 1.8	0.74
N-3 fatty acids (g/d)	0.32 ± 0.04	0.26 ± 0.03	0.33

^a^Data are presented as means ± SE

^b^Obtained from paired t-test for comparison of two intervention groups

All randomly assigned individuals were included in the ITT analysis (n = 47). The effects of sumac supplementation on anthropometric measures and blood pressure of the study participants included in the ITT analysis are shown in Table 3. Sumac supplementation has decreased systolic blood pressure by 5 mmHg (128.8 ± 21.4 at the baseline vs. 123.2 ± 17.6 after 6 weeks intervention, P = 0.001). In placebo group, a significant decrease was seen in waist circumference (107.6 ± 10.2 at the baseline vs. 106.5 ± 10.3 after 6 weeks, P = 0.01). The comparison of changes in two trial arms showed that sumac supplementation has significantly declined systolic blood pressure (sumac group -5.59 ± 10.6 vs. control group 0.76 ± 10.5, *P* = 0.004), but did not change anthropometric indices or diastolic blood pressure.

**Table 3:** The effects of sumac supplementation on anthropometric measures and blood pressure of adults with metabolic syndrome (with intention to treat analysis) (*n* = 47)^a^

	Sumac group^b^				Control group^c^				
	Before	6^th^ week	Change^d^	P^e^	Before	6^th^ week	Change^d^	P^e^	P^f^
Weight (kg)	79.6 ± 14.3	79.2 ± 14.2	-0.45 ± 1.9	0.11	79.6 ± 14.4	79.3 ± 14.2	-0.27 ± 0.98	0.06	0.56
BMI (kg/m^2^)	31.5 ± 4.6	31.3 ± 4.5	-0.18 ± 0.83	0.14	31.3 ± 4.6	31.5 ± 4.5	-0.09 ± 0.42	0.13	0.52
Waist circumference (cm)	106.9 ± 11.7	106.5 ± 10.8	-0.40 ± 3.4	0.41	107.6 ± 10.2	106.5 ± 10.3	-1.1 ± 2.9	0.01	0.34
Systolic blood pressure (mmHg)	128.8 ± 21.4	123.2 ± 17.6	-5.59 ± 10.6	0.001	127.5 ± 18.8	128.2 ± 18.6	0.76 ± 10.5	0.62	0.004
Diastolic blood pressure (mmHg)	83.2 ± 10.2	82.1 ± 10.6	-1.07 ± 6.36	0.25	83.1 ± 1.4	83.5 ± 1.7	0.36 ± 6.48	0.70	0.19

^a^Reported values are means ± SD

^b^In the sumac group, participants received sumac capsules (500 mg/twice daily) for 6 weeks

^c^In the control group, participants received lactose capsules (500 mg/twice daily) for 6 weeks

^d^Calculated by subsidizing baseline values from values of 6^th^ week

^e^Obtained from paired t-test for comparison of with-in group differences

^f^Obtained from paired t-test for comparison of between-group differences

The impact of sumac supplementation on glycemic indices and lipid profiles of the study participants included in the ITT analyses is presented in Table 4. In sumac group, no significant difference was found in glycemic or lipid profiles. But in control group, a significant increase in total cholesterol was seen (159.6 ± 34.7 at the baseline vs. 168.5 ± 44.4 after 6 weeks, *P* = 0.01). Additionally, marginally significant increases in serum triglyceride (170.9 ± 11.5 at the baseline vs. 185.9 ± 97.4 after 6 weeks, *P* = 0.07) and LDL-c (80.9 ± 30.3 at the baseline vs. 86.6 ± 30.1 after 6 weeks, *P* = 0.07) was found in control group. However, when we compared changes in sumac and control group, no significant differences in glycemic or lipid profiles were observed.

**Table 4:** The effects of Sumac supplementation on features of glycemic and lipid profile of adults with metabolic syndrome (with intention to treat method) (*n* = 47)^a^

	Sumac group^b^				Control group^c^				
	Baseline	6^th^ week	Change^d^	P^e^	Baseline	6^th^ week	Change^d^	P^e^	P^f^
FBS (mg/dL)	108.7 ± 30.9	108.0 ± 28.9	-0.48 ± 13.4	0.80	108.2 ± 24.2	107.4 ± 25.4	-0.74 ± 17.3	0.77	0.94
Serum triglyceride (mg/dL)	177.0 ± 10.6	173.0 ± 10.36	-4.00 ± 40.5	0.50	170.9 ± 11.5	185.9 ± 97.4	14.9 ± 56.7	0.07	0.10
Total cholesterol (mg/dL)	165.9 ± 5.6	165.5 ± 5.5	-0.44 ± 24.5	0.90	159.6 ± 34.7	168.5 ± 44.4	9.1 ± 23.7	0.01	0.12
HDL-c (mg/dL)	44.5 ± 10.32	45.00 ± 11.4	0.51 ± 10.7	0.74	44.23 ± 10.5	44.80 ± 9.9	0.57 ± 10.3	0.70	0.98
LDL-c (mg/dL)	86.0 ± 34.3	85.9 ± 32.0	-0.16 ± 26.5	0.97	80.9 ± 30.3	86.6 ± 35.1	5.6 ± 20.4	0.07	0.34

^a^Reported values are means ± SD

^b^In the sumac group, participants received sumac capsules (500 mg/twice daily) for 6 weeks

^c^In the control group, participants received lactose capsules (500 mg/twice daily) for 6 weeks

^d^Calculated by subsidizing baseline values from values of 6^th^ week

^e^Obtained from paired t-test for comparison of with-in group differences

^f^Obtained from paired t-test for comparison of between-group differences

In the per-protocol analysis (*n* = 32), a significant reduction in systolic blood pressure was found in sumac group (129.0 ± 23.2 at the baseline vs. 122.1 ± 19.4 after 6 weeks, *P* = 0.002), as shown in Table 5. In control group, waist circumference was significantly declined (108.3 ± 10.7 at the baseline vs. 106.9 ± 11.9 after 6 weeks, *P* = 0.03). When we compared changes in two groups, we found that sumac supplementation led to a significant reduction of systolic blood pressure (sumac group -6.9 ± 11.71 vs. control group 2.06 ± 11.74, *P* = 0.01). As shown in Table 6, in per-protocol analysis, we found that sumac supplementation did not affect glycemic and lipid profile of participants. But in control group, a slight increase in serum triglycerides (171.5 ± 90.1 at the baseline vs. 195.4 ± 113.5 after 6 weeks, *P* = 0.05) and a significant rise in total cholesterol (158.9 ± 32.0 at the baseline vs. 171.88 ± 44.0 after 6 weeks, *P* = 0.01) were seen. Comparison of changes in two trial arms revealed that sumac supplementation could prevent a marginally significant rise in serum triglycerides (sumac group -4.9 ± 48.7 vs. control group 23.9 ± 66.7, *P* = 0.09).

**Table 5:** The effects of Sumac supplementation on anthropometric measures and blood pressure of adults with metabolic syndrome (with per protocol method) (*n* = 32)^a^

	Sumac group^b^				Control group^c^				
	Baseline	6^th^ week	Change^d^	P^e^	Baseline	6^th^ week	Change^d^	P^e^	P^f^
Weight (kg)	80.9 ± 15.8	80.5 ± 15.7	-0.31 ± 1.3	0.16	81.11 ± 15.8	80.7 ± 15.7	-0.35 ± 1.17	0.10	0.91
BMI (kg/m^2^)	31.7 ± 4.6	31.6 ± 4.6	-0.11 ± 0.48	0.18	31.9 ± 4.7	31.7 ± 4.6	-0.14 ± 0.48	0.10	0.82
Waist circumference (cm)	107.5 ± 12.7	107.9 ± 11.7	-0.50 ± 3.83	0.46	108.3 ± 10.7	106.9 ± 11.9	-1.39 ± 3.38	0.03	0.40
Systolic blood pressure (mmHg)	129.0 ± 23.2	122.1 ± 19.4	-6.90 ± 11.71	0.002	127.1 ± 18.5	129.2 ± 20.1	2.06 ± 11.74	0.33	0.01
Diastolic blood pressure (mmHg)	83.3 ± 9.4	82.1 ± 10.6	-1.28 ± 7.34	0.33	83.1 ± 9.5	83.6 ± 11.1	0.65 ± 6.87	0.59	0.15

^a^Reported values are means ± SD

^b^In the sumac group, participants received sumac capsules (500 mg/twice daily) for 6 weeks

^c^In the control group, participants received lactose capsules (500 mg/twice daily) for 6 weeks

^d^Calculated by subsidizing baseline values from values of 6^th^ week

^e^Obtained from paired t-test for comparison of with-in group differences

^f^Obtained from paired t-test for comparison of between-group differences

**Table 6:** The effects of Sumac supplementation on features of glycemic and lipid profile of adults with metabolic syndrome (with per protocol method) (*n* = 32)^a^

	Sumac group^b^				Control group^c^				
	Baseline	6^th^ week	Change^d^	P^e^	Baseline	6^th^ week	Change^d^	P^e^	P^f^
FBS (mg/dL)	109.4 ± 34.9	107.9 ± 32.6	-1.53 ± 15.83	0.58	107.2 ± 26.0	106.7 ± 27.7	-0.46 ± 20.93	0.90	0.82
Serum triglyceride (mg/dL)	179.8 ± 83.7	175.8 ± 81.2	-4.09 ± 48.75	0.63	171.5 ± 90.1	195.4 ± 113.5	23.93 ± 66.76	0.05	0.09
Total cholesterol (mg/dL)	166.7 ± 36.5	165.2 ± 32.1	-1.46 ± 27.84	0.76	158.9 ± 32.0	171.8 ± 44.0	12.90 ± 27.70	0.01	0.10
HDL-C (mg/dL)	43.2 ± 8.6	44.3 ± 10.9	1.09 ± 12.66	0.63	42.1 ± 9.3	43.6 ± 8.2	1.53 ± 11.96	0.47	0.89
LDL-C (mg/dL)	87.5 ± 31.4	85.8 ± 25.5	-1.74 ± 30.10	0.76	82.5 ± 27.4	89.1 ± 32.3	6.58 ± 23.97	0.13	0.33

^a^Reported values are means ± SD

^b^In the sumac group, participants received sumac capsules (500 mg/twice daily) for 6 weeks

^c^In the control group, participants received lactose capsules (500 mg/twice daily) for 6 weeks

^d^Calculated by subsidizing baseline values from values of 6^th^ week

^e^Obtained from paired t-test for comparison of with-in group differences

^f^Obtained from paired t-test for comparison of between-group differences

## Discussion

The current cross-over RCT revealed that sumac supplementation has significantly decreased systolic blood pressure among adults with MetS. Sumac supplementation might also prevent a significant rise in serum triglyceride among study subjects. This was the first cross-over trial that examined the effect of sumac supplementation on MetS markers in both men and women with MetS.

Previous evidence has shown that having MetS is drastically related to increased risk of nonalcoholic fatty liver, steatohepatitis [26], stroke [27], Alzheimer [28], cardiovascular morbidity and mortality [29], progression of diabetic nephropathy [30] and some cancers [31]. Considering the high prevalence of MetS, its complications, expensive drugs therapy and their interactions and side effects, finding a safe alternative treatment is worth. Our findings suggested that adjuvant therapy with sumac could be used for MetS treatment and decreasing its subsequent comorbidities.

Similar to our study, Asgary et al. [1] examined the effect of sumac supplementation (500 mg/twice a day) on cardiovascular risk factors in patients with dyslipidemia in a cross-over RCT. Duration of each phase was 4 weeks with a 2-week washout period between them. They showed favorable effect of sumac supplementation on BMI, SBP, DBP, and total cholesterol and flow-mediated dilation (FMD) in hyperlipidemic patients. In a placebo-controlled parallel trial, overweight and obese subjects consumed 500 mg sumac twice a day for six weeks. Although weight, waist circumference, BMI and insulin resistance were significantly decreased, there were no significant changes in FBG and leptin concentrations [24]. Furthermore, Shidfar et al. [10] investigated the effect of 3 g/day sumac powder on serum glycemic profile, ApoB, ApoA-I and total antioxidant capacity in 41 patients with type 2 diabetes in a 3-month parallel RCT (sumac group, *n* = 22; placebo group, *n* = 19). They documented favorable effects of sumac on glycemic status, apoB, apoA-I and total antioxidant capacity (TAC). Additionally, in this trial they found desirable effect of sumac on C-reactive protein (hs-CRP), MDA, paraoxonase 1 (PON1) activity and HOMA-IR [4]. Another RCT which was conducted on adolescents (aged 12–18 years) demonstrated that 500 mg sumac supplementation three times a day, for 4 weeks, could decrease serum TG, LDL-c and total cholesterol values. However, there was no significant change in HDL-c [23]. A systematic review and meta-analysis published in 2018 has investigated the effect of sumac on total cholesterol, HDL-c, LDL-c and triglyceride and did not show any significant difference between intervention and control groups. This meta-analysis reported that definite conclusions could not be found, due to insufficient eligible RCTs [22]. This point must be taken into account that the previous publications revealed contradictory results, due to different study design, health status of the study participants, prescribed energy intake during the intervention, and duration of the interventions.

Previous studies suggested some pathways to explain the mechanisms for beneficial effects of sumac on MetS components. Sumac extract is a good source of natural antioxidants. Oxidative stress could lead to hypertension by two pathways including, increasing vascular contractile activity through damaging the endothelium [32] and narrowing vascular lumen by stimulating proliferation and hypertrophy of vascular smooth muscle and collagen deposition [33]. Antioxidative phenolic components of sumac, such as tannins and flavonoids [34], would have favorable effect on blood pressure. Moreover, polyphenols with high resin-binding capacities could influence the gastrointestinal tract and reduce the lipid absorption. Additionally, high amount of water soluble tannins in sumac have important role in its antioxidant activity [35]. Sumac could also have lowering effect on serum cholesterol through the inhibiting the xanthine oxidase. Antioxidant and radical-scavenging activities of sumac against the lipid peroxidation could benefit lipid profiles [36]. Sumac supplementation could also be an effective treatment for obesity due to decreasing the absorption of food lipids through inhibiting the pancreatic lipase enzyme [37, 38]. This herb could decrease the digestion and absorption of carbohydrates by inhibiting α-amylase [39, 40], α-glucosidase [40] and glucose transporter-2 (GLUT-2) [41] in intestine. Furthermore, sumac supplementation could influence the insulin secretion and insulin action, although no effect on glucose transporter-4 (GLUT-4) genes expression was found [39].

In the current investigation, there are some points that strengthen this RCT. The study was conducted on both male and female population. The cross-over design of the study resulted in independent relations from personal features or genetic variables. Moreover, performing ITT analysis helped us to include all subjects in the analysis. Additionally, triple-blinded design of the trial would decrease risk of bias. Nevertheless, some limitations must be kept in mind. High prevalence of covid-19 pandemic and the traffic restrictions for this pandemic during the study implementation increased loss to follow-up of participants. Furthermore, there was no biomarker to evaluate compliance of individuals to the sumac supplementation. Additionally, because of low number of participants we could not perform stratified analysis by gender.

## Conclusion

This cross-over randomized controlled trial revealed that sumac supplementation could decrease systolic blood pressure and might prevent a rise in triglyceride concentration in men and women with metabolic syndrome. Daily intake of 1000 mg sumac, as an adjuvant therapy, could be beneficial in management of MetS in adults.

## Data Availability

The data that support the findings of this study are available from the corresponding author (PS) upon reasonable request.

## Abbreviations

MetS: Metabolic syndrome
CVD: Cardiovascular disease
T2DM: Type II diabetes mellitus
RC: Rhus Coriaria
RCT: Randomized clinical trial
MDA: Malondialdehyde
HOMA-IR: Homeostatic model assessment of insulin resistance
hs-CRP: High sensitive C-reactive protein
IEMRC: Isfahan Endocrine and Metabolic Research Center
TG: Triglyceride
SBP: Systolic blood pressure
DBP: Diastolic blood pressure
FBS: Fasting blood sugar
ITT: Intention-to-treatment
WC: Waist circumference
BMI: Body mass index
MET: Metabolic equivalent
LOCF: Last-observation-carried-forward
FMD: Flow-mediated dilation
PON1: Paraoxonase 1
NCEP/ATP III: National Cholesterol Education Program Adult Treatment Panel III
HDL-c: High density lipoprotein-cholesterol
LDL-c: Low density lipoprotein-cholesterol
TAC: Total antioxidant capacity
GLUT-2: Glucose transporter-2
GLUT-4: Glucose transporter-4
